# COVID-19-Associated Pulmonary Aspergillosis in a Tertiary Hospital

**DOI:** 10.3390/jof8020097

**Published:** 2022-01-19

**Authors:** García-Clemente Marta, Forcelledo-Espina Lorena, Martínez-Vega Laura, Lanza-Martínez Angela, Leoz-Gordillo Blanca, Albillos-Almaraz Rodrigo, Solís-García Marta, Melón-García Santiago, Pérez-Martínez Liliana, Sánchez-Nuñez Maria Luisa, Peláez-García de la Rasilla Teresa

**Affiliations:** 1 Pneumology Department, Central Universitary Hospital of Asturias (HUCA), 33011 Oviedo, Spain; lauramarve@gmail.com (M.-V.L.); kalaka94@hotmail.com (L.-M.A.); msolis1995@gmail.com (S.-G.M.); lila_jkr@hotmail.com (P.-M.L.); 2 ICU Department, HUCA, 33011 Oviedo, Spain; lforcelledoespina@yahoo.es (F.-E.L.); blanca.leoz@gmail.com (L.-G.B.); rodrigoalbillos@gmail.com (A.-A.R.); 3 Microbiology Department, HUCA, 33011 Oviedo, Spain; santiago.melon@sespa.es (M.-G.S.); mtpelaez@gmail.com (P.-G.d.l.R.T.); 4 Hospital Direction, HUCA, 33011 Oviedo, Spain; marisasanchezn@sespa.es

**Keywords:** COVID-19, Aspergillosis, CAPA, mortality

## Abstract

Our study aims to assess the prevalence of CAPA (COVID-19-associated pulmonary aspergillosis) and describe the associated risk factors and their impact on mortality. A prospective study was conducted. We included patients with COVID-19 disease who were admitted to the ICU with a diagnosis of respiratory failur. Mycological culture and other biomarkers (calcofluor staining, LFD, LFA, PCR, GM, and B-D-glucan) were performed. A total of 300 patients were included in the study. Thirty-five patients were diagnosed with CAPA (prevalence 11.7%). During admission, 57 patients died (19%), and, in the group of CAPA patients, mortality was 31.4%. In multivariate analysis, independent risk factors associated with CAPA diagnosis were age (OR: 1.05; 95% CI 1.01–1.09; *p* = 0.037), chronic lung disease (OR: 3.85; 95% CI 1.02–14.9; *p* = 0.049) and treatment with tocilizumab during admission (OR: 14.5; 95% 6.1–34.9; *p* = 0.001). Factors independently associated with mortality were age (OR: 1.06; 95% CI 1.01–1.11; *p* = 0.014) and CAPA diagnosis during admission (OR: 3.34; 95% CI 1.38–8.08; *p* = 0.007). CAPA is an infection that appears in many patients with COVID-19 disease. CAPA is associated with high mortality rates, which may be reduced by early diagnosis and initiation of appropriate antifungal therapy, so screening of COVID-19 ARDS (acute respiratory distress syndrome) patients for CAPA is essential.

## 1. Introduction

The association between influenza virus infection and invasive pulmonary aspergillosis (IPA) has been described in the last decade in patients admitted to intensive care units, with mortality reported as high as 50% in some studies [1,2,3]. The outbreak of the SARS-COV-2 infection has also caused a progressive increase in the number of cases of IPA associated with COVID-19 disease (CAPA), with significant mortality rates [4].

We face significant challenges when diagnosing IPA associated with viral illnesses in general, and especially to SARS-COV-2 infections. Risk factors related to CAPA can be different from those typically reported for IPA infections. In addition, patients usually present severe radiological lesions other than those characteristics of fungal infection. For these reasons, the commonly used IPA diagnostic criteria (EORTC/MSGERC) [5] are not helpful when assessing IPA in these patients.

When clinical and radiological deterioration happens in patients with SARS-COV-2 infection, a progression of ARDS (acute respiratory distress syndrome) or bacterial superinfections are frequently suspected. Usually, changes in antibiotic treatment are made without evaluating other possible diagnoses [6,7]. Even proven *Aspergillus* isolates in microbiological cultures of respiratory samples are sometimes considered colonizers, despite their potential to cause invasive disease. Therefore, since anticipating the diagnosis and treatment can improve patient outcomes [1], it is essential to establish clear diagnostic criteria [5,6,7].

Recently, consensus criteria have been published to define CAPA [8] with microbiological standards, clinical features, and imaging data, allowing clinicians to improve the diagnostic process, classify patients more uniformly, and report results better. However, we continue to face important diagnostic challenges [4,9].

Regarding risk factors associated with CAPA, different articles have described a relationship with treatment with azithromycin, corticosteroids, anti-IL6 drugs, as well as with underlying lung disease [4,10,11,12,13]. To identify apparent risk factors and establish prevention strategies, it is necessary to carry out more extensive studies. An increase in mortality rates has also been described in different single-center cohorts [4,10,11], and again, more comprehensive studies are needed to elucidate the role of CAPA in mortality in patients with acute respiratory failure due to COVID-19.

Our study aimed to assess the prevalence of CAPA in patients with COVID-19 admitted to our hospital’s intensive care unit (ICU) during the first and second waves of the pandemic, describe the associated risk factors, and analyze its impact on the mortality of these patients.

## 2. Materials and Methods

### 2.1. Study Design

A prospective study was conducted at a tertiary university hospital in Asturias (Spain) during the COVID-19 pandemic between 1 March and 31 December 2020. We included patients admitted consecutively to the ICU from the hospitalization ward with a diagnosis of respiratory failure.

Demographic data, comorbidities, chronic pulmonary disease (COPD, asthma, and bronchiectasis), risk factors for invasive fungal infection, microbiological features, radiological data, treatments received in the hospitalization ward and the ICU, and clinical course and mortality were prospectively collected, and mortality was also analyzed three months after discharge. Bolus corticosteroids were administered with 6-methyl prednisolone at a dose of 125 mg daily for three days. The study was in accordance with the Helsinki declaration and national ethical standards. The hospital research ethics committee approved the study protocol.

### 2.2. Microbiology Data Collection

Starting in March 2020, institutional recommendations were to screen patients in ICUs for fungal infections by means of:

1. Fungal cultures from respiratory samples on Sabouraud dextrose agar plates (BioMerieux, Mercy, L’Etoile, France). Fungus identification was performed by a matrix-assisted laser desorption ionization-time of flight (MALDI-TOF) mass spectrometry instrument (Bruker, Spain), following the manufacturer’s instructions.
2. The two commercially manufactured lateral flow devices (LFDs) (AspLFD, OLM Diagnostics, Newcastle upon Tyne, UK) and Lateral Flow Assays (LFAs) (IMMY sona *Aspergillus* Galactomannan Lateral Flow Assay, IMMY, Norman, OK, USA) tests, with a visual reader which provides a semiquantitative reading and removes subjectivity when interpreting results.
3. Quantitative real-time PCR for *Aspergillus* genus as follows: DNA extraction for PCR analysis was performed on an ELITe InGenius automated platform as well as RT-PCR using the *Aspergillus* spp. ELITe MGB kit (Elitegroup, Palex, Spain). The DNA was extracted from a 1-mL volume of BAL fluid and was eluted in a 200-μL saline solution before DNA amplification in the same platform. RT-PCR for *Aspergillus* genus was performed by an *Aspergillus* spp. ELITe MGB kit, which was CE-in-vitro diagnostic (CE-IVD) validated on a diverse range of sample types. The target region was the ribosomal DNA18S (rDNA18S), and the human B-globin gene was used as an internal standard. The fungal DNA copy number was expressed as copies/mL in relation to a rDNA18s standard curve.
4. GM testing was performed using Platelia™ *Aspergillus* (Bio-Rad Laboratories) with a cut-off value of ≥0.5 in serum and ≥1.0 in BAL or ≥4 in tracheal and bronchial aspirates.
5. Detection of 1,3-β-d-glucan (βDG) in serum was performed with the Wako β-glucan test (Fujifilm Wako Pure Chemical Corporation). A cut-off value of 7 pg/mL was used.
6. Antifungal susceptibility to itraconazole, posaconazole, voriconazole, and isavuconazole was determined using the colorimetric broth microdilution Sensititre Yeast-One (SYO; Trek Diagnostic System, Cleveland, OH, USA) method as well as the agar diffusion Etest (bioMérieux, Marcy l’Etoile, France) method.

Our epidemiological cutoff values (ECVs) of triazole resistance were defined following the criteria published by the CLSI [14,15] and as proposed by Espinel-Ingroff et al. [16]. Isolates showing an MIC above the ECVs were considered non-wild-type.

### 2.3. CAPA Case Definitions

A diagnosis of COVID-19-associated pulmonary aspergillosis (CAPA) was defined based on the 2020 European Confederation of Medical Mycology/International Society for Human and Animal Mycology (ECCM/ISHAM) [8] consensus criteria, and according to these criteria, patients were classified as proven CAPA, probable CAPA, possible CAPA or no evidence of CAPA. CAPA diagnosis was defined as the earliest date when a diagnostic feature was identified.

The characteristics of the patients diagnosed with CAPA during their evolution were evaluated, comparing them with those from patients who did not present CAPA during their admission to the ICU.

### 2.4. Statistical Study

A descriptive analysis was performed. Proportions or means with standard deviation (SD) described the patient sample. Continuous variables were compared using Student’s T-test, while chi-squared or Fisher exact tests were used for categorical dependent data analysis, as appropriate. Baseline characteristic differences that were statistically significant in the univariate analysis were tested in multivariate binary logistic regression models to define independent risk factors associated with CAPA diagnosis. Independent risk factors associated with mortality were also analyzed. All tests were two-sided, and a *p*-value < 0.05 was considered statistically significant. Statistical analysis was performed using SPSS version 20.0. CAPA prevalence was calculated by dividing the number of patients diagnosed with CAPA (according to the criteria stated above) divided by the total number of patients diagnosed with COVID-19 disease admitted to the ICU during the first and second waves of the COVID-19 pandemic.

## 3. Results

A total of 300 patients with PCR-confirmed SARS-CoV-2 infection requiring ICU admission due to COVID-19 associated acute respiratory failure were included in the study.

### 3.1. Patients’ Characteristics

We include 300 patients, 28% female, with a mean age of 63.5 ± 11.9 (15–83) years. The median time from ICU admission to CAPA diagnosis was six days (IQR 1–27), and the global ICU mean length of stay was 23.9 ± 16.8 (1–100) days. In relation to comorbidities, 90 patients have cardiovascular diseases (30%), 54 (18%) diabetes, 153 (51%) hypertension, 126 (42%) dyslipidemia, 138 (46%) obesity with BMI > 30, and 34 (11.4%) chronic pulmonary disease. Regarding mechanical ventilation, 260 patients (87%) were treated with invasive mechanical ventilation, 21 (7%) with non-invasive mechanical ventilation, 84 (28%) with high-flow oxygen therapy, and three patients (1%) with ECMO. Forty-five patients (15%) received tocilizumab, 243 (81%) systemic corticosteroids, 135 (45%) corticosteroids boluses, and 63 (21%) azithromycin. Fifty-seven patients (19%) died during admission, and 75 (25%) had died three months after discharge. Table 1 shows the characteristics of the patients included in the study.

### 3.2. CAPA Diagnosis

During the first wave, 62 patients were admitted to ICU, and 11 patients were diagnosed with CAPA, with a prevalence of 17.7%. During the second wave, 238 patients were admitted to ICU, and 24 patients were diagnosed with CAPA, with a prevalence of 10.1%. The total number of patients diagnosed with CAPA was 35, with an overall prevalence of 11.7%. Overall, fifty-seven patients (19%) were deceased during admission, while in the group of CAPA patients, mortality was 31.4% (11/35 patients).

### 3.3. Mycological Results

CAPA patients presented a mean viral load of 17.784.946 copies/10^3^ cells (range 16.220–1.246.612.224). The results of the diagnostic tools of patients with CAPA diagnosis were as follows:

1. Mycological culture was positive in 34 patients (97.1%). Of these, 27 (80%) had an infection caused by a single species of *Aspergillus,* while seven patients (20%) presented a disease caused by more than one species of *Aspergillus*. There was no growth in one patient (3%).

Specifically, the distribution of *Aspergillus* species isolated in the different patients was as follows: 19 (55.9%) *Aspergillus fumigatus*; 5 (14.8%) *A. terreus*; 4 (11.8%) *A. fumigatus* + *A. terreus*; 3 (8.8%) *A. niger*; 1 (2.9%) *A. fumigatus + A. niger*; 1 (2.9%) *A. fumigatus* + *A. nidulans*; 1 (2.9%) *A. fumigatus* + *A. terreus* + *A. niger.* Involved *Aspergillus* species are reported in Table 2. All *A. fumigatus* were azole susceptible. Two *A. terreus* and one *A. niger* showed azole resistance.

2. Calcofluor staining was only performed in ten of the 35 patients to avoid aerosol-generating procedures, being positive in all ten patients.
3. The lateral flow devices performed on available respiratory samples were positive in all patients.
4. *Aspergillus* PCR was positive in all patients, with a mean cycle value of 28.7 (range 22.4–36) and a mean fungal load of 153.341 copies/mL (range 169–2.252.000).
5. Serum GM was positive in only 16.7% of patients. The mean value was 0.32 (range 0.03–1).
6. The GM in respiratory samples was positive in 94.3% of the cases, with a mean value of 5.5 (range 0.08–10).
7. Serum β-D-glucan was performed in 11 patients, being positive in all of them, with a mean value of 93.9 pg/mL (range 7–321.5).

The diagnostic methods are detailed in Table 3.

Median time from ICU admission to the first *Aspergillus* isolation was six days (1–27), and 94.3% of patients had invasive mechanical ventilation. Pulmonary aspergillosis was probable in 7 patients (20%) and possible in 28 (80%). Zero patients had histopathologically proven CAPA. Twenty-five *Aspergillus*-positive cultures (8.3% of all patients) did not fulfill the CAPA case definition criteria and were considered colonized with *Aspergillus*.

### 3.4. Comparison of Patients with and without CAPA Univariate Analysis

In univariate analysis, patients with CAPA were older (68.8 ± 8.1 vs. 62.5 ± 12.3; *p* < 0.001), had more frequent chronic lung disease (22.8% vs. 9.8%; *p* = 0.022), and had been treated more frequently with tocilizumab (57.1% vs. 9.4%; *p* < 0.001) and corticosteroid boluses (65.7% vs. 42.3%; *p* = 0.008). We did not find a relationship with BMI, ICU or hospital stay length, or ventilatory support. We did not find either a link with the use of azithromycin or corticosteroids at doses of 6 mg of dexamethasone, being the usual regimen from the second wave of the pandemic. Table 4 shows the factors associated with CAPA diagnosis.

### 3.5. Comparison of Patients with and without CAPA Multivariate Analysis

In multivariate analysis (Table 5), independent risk factors associated with CAPA diagnosis were age (OR: 1.05; 95% CI 1.01–1.09; *p* = 0.037), chronic lung disease (OR: 3.85; 95% CI 1.02–14.9; *p* = 0.049) and treatment with tocilizumab during admission (OR: 14.5; 95% 6.1–34.9; *p* = 0.001). The use of corticosteroids boluses was not an independent factor associated with CAPA diagnosis when pooled in the multivariate models.

### 3.6. Mortality

Regarding mortality, patients with CAPA had a 31.4% mortality rate during admission, compared to 17.3% in the group of patients without CAPA (*p* = 0.048). Three months after discharge, mortality was 40% in patients with CAPA diagnosis versus 23% in those who had not developed CAPA during admission (*p* = 0.029).

When the mortality of all patients was globally analyzed, 57 patients (19%) died. Factors independently associated with mortality were age (OR: 1.06; 95% CI 1.01–1.11; *p* = 0.014) and CAPA diagnosis during admission (OR: 3.34; 95% CI 1.38–8.08; *p* = 0.007).

## 4. Discussion

Our study reports a CAPA prevalence of 11.7% with a mortality of 31.4%, significantly higher than the 19% observed in patients not diagnosed with CAPA. In the analysis of factors associated with CAPA diagnosis, we describe that these patients were older, had chronic lung disease more frequently, and received treatment with tocilizumab in a higher proportion than those who did not present CAPA.

According to the different studies published, CAPA prevalence rates are highly variable, ranging between 3 and 33% [4,6,17,18,19]. These differences can be attributed to different thresholds for clinical evaluation, diagnostic methods, and differences in criteria for CAPA definition [6,20]. Our data are similar to those published by Prattes et al. [4] in a multicenter study, in which the median prevalence was 10.7%, compared to the 11.7% observed in our study.

Regarding mortality of patients with CAPA, in a prospective study of 108 patients with SARS-CoV-2 pneumonia and ARDS, a higher mortality rate was observed in patients with CAPA than those who did not present aspergillosis (44% vs. 19%). In this study, CAPA diagnosis was associated with ICU mortality in a logistic regression model, even after adjustment for age, need for renal replacement therapy, and sequential organ failure assessment (SOFA) score at ICU admission [8]. In the multicenter study by Prattes et al. [4], the authors reported a reduced survival at 90 days (29% in patients with CAPA vs. 57% in patients without CAPA), remaining as an independent prognostic factor of mortality after adjusting for other variables [4]. Ergün et al. [3] found 53.8% mortality in patients with CAPA compared to 24.1% in patients who did not present CAPA. In our study, the mortality of patients with CAPA during admission was 31.4%, rising to 40% at three months after discharge. We also observed that CAPA diagnosis was an independent mortality risk factor in patients admitted with severe SARS-COV-2 pneumonia.

The mechanism of fungal infection associated with SARS-COV-2 disease is unclear. One hypothesis could be that the virus produces severe damage to the lung tissue with significant alveolar-interstitial lesions, this causing more probability of coinfections acquired by inhalation, such as fungal disease [21,22]. Another contributing factor may be the severe leukopenia caused by the virus, with a significant decrease in the number of CD4 and CD8 lymphocytes and elevation of interleukins (IL-2R, IL-6, IL-10, TNF-alpha) and other inflammatory markers [23]. A function defect may accompany the decrease in lymphocytes, and severe lymphopenia has been established to predict invasive fungal disease in patients with hematological malignancy [8,24]. The respiratory mucosa damage and secondary mucociliary clearance alteration [21,25] would allow *Aspergillus* to infect the respiratory tract, also avoiding the action of the alveolar macrophages due to dysfunction or immune dysregulation both locally and systemically [8,21,26,27,28], this caused by the COVID-19 disease itself and also by treatments such as corticosteroids and immunosuppressants.

Different risk factors associated with CAPA diagnosis have been reported across the different published studies. In the multicenter study by Prattes et al. [4], CAPA diagnosis was associated with older age, with any form of invasive support, and with treatment with anti-IL-6 (tocilizumab) (HR: 2.45; 95% CI 1.41–4.25). These data are similar to those obtained in our study. We also observed a relationship with older age and treatment with tocilizumab as factors associated with isolation of *Aspergillus*. When we analyzed the use of invasive respiratory support in our study, 94% of patients with CAPA were receiving invasive mechanical ventilation. Still, 87% of the overall sample received invasive support; no relationship was observed with CAPA diagnosis.

Although steroid treatment has been linked to the appearance of IPA in patients with influenza [29], it is not clear if it is also associated with SARS-COV-2 infections. In our study, we observed a relationship between the use of boluses of corticosteroids and CAPA in the univariate analysis, but this was not confirmed as an independent risk factor in the multivariate analysis. Similarly, in the series by Prattes et al. [4], a relationship with steroid treatment was not found. However, we must consider that most patients in our study belong to the second wave of the pandemic, when as a usual clinical practice, all patients with respiratory failure received dexamethasone at a dose of 6 mg daily, making it difficult to discriminate the possible effect of corticosteroids on the appearance of CAPA. Still, dexamethasone treatment and anti-IL-6 strategies might also result in increased susceptibility to superinfections [30,31], including CAPA [32], and could lead to increases in CAPA incidence, emphasizing the need for clinical guidelines.

Another factor that should be considered is previous chronic lung damage. In the study published by Prattes et al. [4], chronic lung damage was described as a risk factor for the appearance of CAPA. Our research also observed that this relationship remained an independent risk factor for CAPA diagnosis in multivariate analysis. It is possible that previous colonization by *Aspergillus* in the lungs with the chronic disease could lead to invasive aspergillosis by adding the immune dysregulation and immunosuppression derived from severe SARS-COV-2 disease. It is worth highlighting that, in our study, CAPA was diagnosed after a median of 6 days since admission to the ICU, which would support that previously colonized patients may be more susceptible to present CAPA. Other studies have found similar data, such as Bartoletti et al. [10], with a mean of 4 days (SD:4), Dellière et al. [12], with a mean of 6 days (SD: 10), Fekkar et al. [33], with a median of 7 days (IQR: 2–56), and Lewis White [11], with a median of 8 days (IQR 0–35) from ICU admission to CAPA diagnosis.

A year before the pandemic in our hospital, a diagnostic algorithm for detecting cases of invasive aspergillosis was developed within an antifungal optimization program, based on existing guidelines and available diagnostic tools (calcofluor staining mycological culture, LFD, LFA, PCR, GM, and B-D-glucan). In March 2020, this algorithm was adapted to detect existing CAPA cases in intensive care units. Therefore, mycological culture and all biomarkers mentioned above were performed on all respiratory samples of SARS-COV-2 patients admitted to ICUs. [34,35,36,37,38,39,40]. When we analyzed these diagnostic tests, both mycological culture and fungal biomarkers demonstrated high sensitivity and specificity in the diagnosis of CAPA patients, with the significant exception of GM detection in serum, which had a sensitivity as low as 16.7%. The initial difficulties in diagnosing CAPA have had a lot to do with the low sensitivity of serum biomarkers [10,11,35] and the reluctance at the beginning of the pandemic to perform bronchoscopies due to the risk of aerosol exposure and the spread of the disease, which has led to confusing literature [41,42,43,44]. In general, the first cases were reported in early 2020, in the form of case reports or small series of cases [4,45,46,47] and obtaining diagnoses by bronchial aspirates, making it difficult to differentiate invasive aspergillosis from what may be colonization in a group of severe patients with acute respiratory distress syndrome with clinical and radiological alterations superimposable to the picture of fungal infection [3,48]. Detection of *Aspergillus* in upper respiratory tract specimens, such as sputum or tracheal aspirate, often does not distinguish between *Aspergillus* colonization and invasive disease.

It is essential to keep in mind that, although non-culture-based methods, such as LFD, LFA, PCR, GM are not validated for respiratory specimens other than BAL, we believe that it is crucial, in line with other authors [11,49,50,51], to “use all the available cards in the deck,” i.e., use the available patient samples (NBL, TA) to detect CAPA cases in the first sample tested. Otherwise, there will continue to be a clear under-diagnosis of aspergillosis in COVID-19 patients, significant variability in incidence, delayed initiation of therapy, and subsequently increased patient morbidity and mortality. This delay has been significantly associated with mortality among intubated patients with COVID-19: White et al. [11] reported significantly higher mortality rates in those with fungal diseases (53% vs. 32%; *p* = 0.04), which was explicitly driven by patients with fungal infection who were not receiving antifungal therapy (90% mortality), whereas mortality was significantly (*p* = 0.008) reduced to 38.5% in those with the fungal disease who received antifungal therapy.

It would be essential to determine if antifungal treatment in patients with positive tests for *Aspergillus* in respiratory samples leads to better outcomes. A proven invasive Aspergillosis option would be voriconazole or isavuconazole [8,52]. Similarly, given the high prevalence of *Aspergillus* in respiratory samples from patients with COVID-19 admitted to ICU, it would be important to determine the use of antifungals as prophylaxis or preventive treatment. There are some ongoing trials on this topic (ClinicalTrials.gov (accessed on 20 December 2021) identifier: NCT04707703). In some centers, the use of inhaled amphotericin B is evaluated when a positive GM is observed in lower respiratory samples, even though the PCR and culture are negative [52].

We must acknowledge some limitations in our study, such as being performed in a single hospital center, making it difficult to extrapolate the data to other populations. Another limitation may be that no patient has a proven API, given the difficulty in taking histological samples in this type of patient. However, the number of patients included in the study is high, which may be a strength.

## 5. Conclusions

In conclusion, large series have been published, and different cohorts [10,11,34,53,54,55] have been followed. CAPA is now considered an infection that appears in a considerable number of patients with COVID-19 disease [4,40]. This disease is associated with high mortality rates, which may be reduced by early diagnosis and initiation of appropriate antifungal therapy, so screening COVID-19 ARDS patients for CAPA is essential. There is, therefore, a need for a strategic approach for the diagnosis and subsequent management of CAPA using all the available samples and tools.

## Figures and Tables

**Table 1:** Characteristics of the patients included in the study.

Characteristic	N = 300
Age (years)	63.5 ± 11.9 (15–83)
Female, n (%)	84 (28%)
BMI (Kg/m^2^)	30.7 ± 5.1 (18–49)
Ward length of stay before ICU (days)	2.5 ± 3.1 (0–25)
ICU length of stay (days)	23.9 ± 16.8 (1–100)
Hospital length of stay (days)	39.1 ± 25.7 (7–147)
Time from ICU admission to CAPA diagnosis (days, median)	6 (IQR 1–27)
Comorbidities n (%)	
Cardiovascular disease	90 (30%)
Diabetes	54 (18%)
Hypertension	153 (51%)
Dyslipidemia	126 (42%)
Obesity BMI > 30	138 (46%)
Chronic pulmonary disease	34 (11.4%)
Invasive mechanical ventilation	260 (87%)
Non-invasive mechanical ventilation	21 (7%)
High-flow oxygen therapy	84 (28%)
ECMO	3 (1%)
Treatment (%)	
Azithromycin	63 (21%)
Tocilizumab	45 (15%)
Systemic Corticosteroids	243 (81%)
Systemic Corticosteroids boluses	135 (45%)
Death during admission	57 (19%)
Death at three months	75 (25%)

**Table 2:** *Aspergillus* species isolated in CAPA patients.

*Aspergillus* Species	N = 34	%
*A. fumigatus*	19	55.9%
*A. terreus*	5	14.8%
*A. fumigatus* + *A. terreus*	4	11.8%
*A. niger*	3	8.8%
*A. fumigatus* + *A. nidulans*	1	2.9%
*A. fumigatus* + *A. niger*	1	2.9%
*A. fumigatus* + *A. terreus* + *A. niger*	1	2.9%

**Table 3:** Diagnostic methods.

CAPA CASES	BAL Positive/BAL PERFORMED	TA Positive/TA Performed
35	CULTURE 7/8	CULTURE 34/35
	LFD 7/8	LFD 35/35
	PCR 7/8	PCR 35/35
	GM 7/8	GM 35/35

CAPA: COVID-19-associated pulmonary aspergillosis; BAL: bronchoalveolar lavage; TA: tracheal aspirate; LFD: lateral flow device; PCR: polymerase chain reaction; GM: Galactomannan.

**Table 4:** Factors associated with CAPA diagnosis. Univariate analysis.

Characteristic	N = 300	No CAPA (n = 265)	CAPA (n = 35)	*p*
Age (years)	63.5 ± 11.9 (15–83)	62.5 ± 12.3	68.8 ± 8.1	<0.001
Female gender n (%)	84 (28%)	74 (27.9%)	10 (28.6%)	0.903
BMI (Kg/m^2^)	30.7 ± 5.1 (18–49)	30.9 ± 5.2	29.8 ± 4.7	0.277
ICU length of stay (days)	23.9 ± 16.8 (1–100)	23.5 ± 16.9	26.4 ± 15.9	0.348
Hospital length of stay (days)	39.1 ± 25.7 (7–147)	39.2 ± 27.1	38.8 ± 17.1	0.924
Invasive mechanical ventilation (days)	19.3 ± 15.8	19.4 ± 16.7	18.7 ± 10.2	0.730
Comorbidities				
Cardiovascular disease n (%)	90 (30%)	79 (29.7%)	11 (31.4%)	0.844
Diabetes n (%)				
Hypertension n (%)	54 (18%)	45 (17%)	9 (25.7%)	0.206
Dyslipidemia n (%)	153 (51%)	130 (49.1%)	23 (65.7%)	0.064
Obesity BMI > 30	126 (42%)	108 (40.8%)	18 (51.4%)	0.229
n (%)	138 (46%)	126 (47.6%)	12 (34.3%)	0.139
Chronic pulmonary disease n (%)				
	34 (11.4%)	26 (9.8%)	8 (22.8%)	0.022
Invasive mechanical ventilation	260 (87%)	227 (85.7%)	33 (94.3%)	0.158
Non-invasive mechanical ventilation	21 (7%)	18 (6.8%)	3 (8.6%)	0.698
High-flow oxygen therapy	84 (28%)	74 (27.9%)	10 (28.6%)	0.936
ECMO	3 (1%)	3 (1.1%)	0 (0%)	0.527
Treatment				
Azithromycin	63 (21%)	55 (20.8%)	8 (22.9%)	0.774
Tocilizumab	45 (15%)	25 (9.4%)	20 (57.1%)	<0.001
Corticosteroids	243 (81%)	213 (80.4%)	30 (85.7%)	0.449
Corticosteroids boluses	135 (45%)	112 (42.3%)	23 (65.7%)	0.008
Death during admission	57 (19%)	46 (17.3%)	11 (31.4%)	0.048
Death at three months	75 (25%)	61 (23%)	14 (40%)	0.029

**Table 5:** Factors associated with CAPA diagnosis. Multivariate analysis.

Characteristic	OR	CI 95%	*p*
Age	1.05	1.01–1.09	0.037
Chronic pulmonary disease	3.85	1.02–14.9	0.049
Tocilizumab	14.5	6.1–34.9	0.001

## Abbreviations

ARDS	Acute respiratory distress syndrome
BAL	Bronchoalveolar lavage.
B-D-G	Beta-D glucano.
BMI	Body mass index.
CAPA	COVID-19 Associated Pulmonary Aspergillosis)
CI	Confidence interval.
COVID	Coronavirus disease.
ECMM/ISHAM	European Confederation of Medical Mycology/International Society for Human and Animal Mycology
ECMO	Extracorporeal membrane oxygenation.
EORTC/MSGERC	Europena Organisation for Research and Treatment of Cancer/Mycoses Study Group Education and Research Consortium.
GM	Galactomannan.
HR	Hazard ratio
ICU	Intensive care unit.
IL	Interleukin.
IPA	Invasive pulmonary aspergillosis.
IQR	Interquartile range.
LFA	Lateral flow assays
LFD	Lateral flow device.
MALDI-TOF	Matrix-assisted laser desorption ionization-time of -light
NBL	Non-bronchoscopic lavage
OR	Odds ratio
PCR	Polymerase chain reaction.
SD	Standard deviation.
SOFA	Sequential organ failure assessment
TA	Tracheal aspirate.
TNF-alpha	Tumor necrosis factor alpha
